# Relative age effects in Elite Chinese soccer players: Implications of the ‘one-child’ policy

**DOI:** 10.1371/journal.pone.0228611

**Published:** 2020-02-14

**Authors:** Zhen Li, Lijuan Mao, Christina Steingröver, Nick Wattie, Joseph Baker, Jörg Schorer, Werner F. Helsen

**Affiliations:** 1 Department of Physical Education and Sports Training, Shanghai University of Sport, Shanghai, China; 2 Department of Sport and Motion, Institute of Sport Science, University of Oldenburg, Oldenburg, Germany; 3 Faculty of Health Sciences, University of Ontario Institute of Technology, Oshawa, ON, Canada; 4 School of Kinesiology and Health Science, York University, Toronto, ON, Canada; 5 Research Centre for Movement Control and Neuroplasticity, Department of Movement Sciences, KU Leuven, Leuven, Belgium; Middlesex University, UNITED KINGDOM

## Abstract

The relative age effect (RAE) refers to the asymmetrical distribution of birthdates in a cohort found in many achievement domains, particularly in sports with many participants like soccer. Given the uniqueness of the one-child policy in China, this study examined the existence of the RAE in elite Chinese male and female soccer players generally and relative to their playing position on the field. Results showed a clear and obvious RAE for all age groups (U20 male, U18 male, adult female and U18 female) with the observed birthdate distributions for each age group significantly different from expected distributions (p<0.05). Additionally, we noticed a differential RAE according to the players’ position on the field as reflected in different effect sizes. In male players, the RAE was significantly greater in Defenders (DF) and Goalkeepers (GK) compared to Midfielders (MF) and Forwards (FW) (V_DF_ = 0.266>V_GK_ = 0.215>V_MF_ = 0.178>V_FW_ = 0.175). In female players, GKs had a larger RAE (V_GK_ = 0.184>0.17, V_DF_ = 0.143, V_MF_ = 0.127, V_FW_ = 0.116). To reduce the negative consequences associated with RAEs throughout player development systems, potential solutions are discussed.

## Introduction

Soccer governing bodies divide youth players into age categories to provide equal opportunity for participation and competition. However, profiles of athletes’ chronological age relative to the date used to group the players (i.e. the ‘cutoff date’) suggest such categorizations may be unfair to players born late in the selection year [1]. For instance, the widespread use of cutoff dates appears to result in substantial disadvantages in evaluations of strength, speed, weight, and height when relatively younger players are compared to older peers in the same age group [2]. In turn, these differences result in greater opportunities for players born early to access better coaching and higher quality training [1, 3]. The broad and consistent differences that result from the respective birthdates of individuals grouped into the same age group are termed relative age effects (RAEs).

Although first identified in ice hockey [4], RAEs have been observed in many sports, including baseball [5], basketball [6], boxing [7], golf [8], gymnastics [4], handball [9], ice hockey [10, 11], soccer [12], swimming [13], tennis [14], and volleyball [15]. In addition, there is some evidence that RAEs are not consistently reflected across all sporting domains. For instance, there appears to be an opposite result in gymnastics [16], with more gymnasts born close to the end of the selection year due to the advantages of small body size and flexibility. As well, there were no relative age effects in boxing and swimming, arguably because the method used to group athletes (e.g., by body weight and age on the date of competition, respectively) prevents RAEs from occurring [17]. There have also been some interesting findings highlighting that players born later in the cohort, who are initially seen as being disadvantaged, often go on to have greater eventual success in their professional careers compared to those born early [18–20]. All this to say, RAEs are inherently more complex than they appear on the surface.

The sport that has been examined most often for RAEs, and where results have been among the largest and most consistent, is soccer [1]. For instance, RAEs in male soccer have been noted in Belgium [2], England [21], France [22], Germany [23], Japan [24], Spain [25] and Switzerland [26], and have been examined in relation to factors, such as age and sex [27], geographical properties [28], income [29], and playing position [26, 30]. Generally, soccer players who are relatively older are more likely to advance to the next selection of talent identification programs, as well as to attain more advanced coaching and match playtime [3]. Although various hypotheses explaining RAEs have been proposed [1, 31, 32], one of the underlying mechanisms in elite soccer seems to be the talent identification process [25]. In many countries talent identification initiatives aim to discover athletes with greatest potential for success in the preferred sport so that resources can be optimally aligned for development. However, the process of identification is largely determined by physical attributes and, to a lower extent, match experience [33]. As a result, the identification process is biased because these variables are typically better/higher in early maturers [34–36].

Despite the consistency of the RAE in the countries examined to date, the phenomenon has seldom been studied in Chinese sports in general and soccer in particular. Two publications [37, 38] have studied the relationship between the grouping selection system and RAEs in junior soccer players, but there is no reference to the one-child policy. This consideration is, however, important for several reasons. First, very little is known about athlete development in China. Second, from 1979 to 2015, China’s “one-child policy” (i.e., that families were restricted to having just a single child) was fundamentally different from other countries. Since the onset of the one-child policy in China, the total fertility rate, defined as the mean number of children born per woman, decreased from 2.9 in 1979 to 1.7 in 2004 [39]. As a result, the number of the national registered children and adolescent students decreased, and, consequently, the total number of sport participants. Specifically, the junior participants of academic schools decreased by 50% from 1996 to 1999 and by another 3.5% from 2002 to 2004, according to population statistics from the Chinese General Administration of Sport [40].

Given this unique context for athlete development, the primary aim of this study was to examine the presence of RAEs in male and female elite Chinese soccer players along with the declination of the population of the junior sport participants in China. Based on prior work in soccer, we hypothesized there would be medium to strong RAEs in this sample. This exploration would extend our understanding of how social and cultural factors affect RAEs. A secondary aim was to examine the relationship between the RAE and the playing position on the field.

## Methods

### Participants

Participants included 2051 Chinese soccer players competing in the 13^th^ China National Championship in 2017 (which included 34 female teams and 37 male teams). Participants were divided into four categories according to regulations formulated by the organization committee. More specifically, they were grouped into female adults, females under the age of 18 years (female U18), males under the age of 18 years (male U18) and males under the age of 20 years (male U20). For the U18 and U20 groups, the cut-off date used for age grouping was the 1^st^ of January. Females in the adult group were over 18 years old. As is common in RAE literature, we collapsed the two-year age bands. This study was approved by the Ethical Committee of the Shanghai University of Sport. All participants provided informed consent to participate in this research.

### Procedure

The Chinese Football Association and the China National Games Organization Committee provided all data. In line with previous research [26], players were categorized into four positions: goalkeeper (GK), defender (DF), midfielder (MF), and forward (FW). In previous studies, RAEs were investigated by looking at differences between the expected and observed number of players born per month or per quarter. Consistent with the selection year, players’ birthdates were categorized into four quartiles (Q), starting with January-March (Q1) and finishing with October-December (Q4).

The observed birthdate distributions of all players were calculated for each quartile. According to previous research, birthdate distributions (e.g., the number of people in quartile 1) were compared against an expected frequency, assuming an equal distribution (e.g. N = 100, expected quartile count = 100/4 = 25) as proposed by Cobley et al. [1]. Chi-square Goodness of Fit tests (X^2^) were used to determine whether the expected and observed birthdate distributions were different from each other. Based on these data, odds ratios (OR) with 95% confidence intervals (95% CI) were calculated between Q1/Q4 as commonly used in RAE studies [1]. The OR for the Q1 vs. Q4 comparison was interpreted as follows: OR<1.22, 1.22≤OR< 1.86, 1.86≤ OR<3.00, and OR≥3.00, indicating negligible, small, medium and large effects, respectively [32]. Pearson’s correlation coefficient was used to assess the association between month of birth and the number of players. In addition, effect sizes were used to assess the X^2^ test results. For the X^2^ analysis, the magnitude of the effect size was measured using f and V. According to Cohen [41] and Cramer [42], for df = 3 (which is the case for all comparisons of birthdate quarters), V = 0.06–0.17 indicated a small effect, V = 0.18–0.29 noted a medium effect, and V>0.30 illustrated a large effect. SPSS version 22.0 was used for all analyses. Statistical significance was set at p<0.05.

## Results

Table 1 shows the distribution of birthdates in male and female elite players by quartile. A strong overrepresentation was found in the U20 male players with 43.18% of players born in the first quartile (X^2^ = 86.98, p< 0.001, OR = 2.19), and similar results in the U18 males with 38.68% of players born in the same quartile (X^2^ = 62.53, p< 0.01, OR = 1.93). As well, there was an overall underrepresentation of male players from the second half of the year (Q3 = 19.75% and Q4 = 19.93%). For the female adult players, the observed distribution was slightly different from the expected distribution (X^2^ = 11.06, df = 3, p<0.05). There was a significant asymmetry in the distribution of female U18 (X^2^ = 29.656, df = 3, p<0.01), as 57% of U18 female players were born in the first half of selection year compared to 43% born in the second half. In total, the observed distributions of all players’ birthdates were significantly different from expected distributions (p<0.01).

**Table 1:** RAEs and birthdate distribution of all soccer players in the 2017^TM^ China National Games.

Age Group	*Birthdate*												*Statistics*			
	Quartile1			Quartile 2			Quartile 3			Quartile 4						
	*Jan*	*Feb*	*Mar*	*Apr*	*May*	*Jun*	*Jul*	*Aug*	*Sep*	*Oct*	*Nov*	*Dec*	X^2^	*p*	OR	Cor
U20 Male**	*106*	*59*	*47*	*31*	*25*	*39*	*27*	*33*	*27*	*35*	*34*	*28*	*86*.*98*	*<0*.*01*	*2*.*19*	*-0*.*65*
	*N = 212 (43*.*18%)*			*N = 95 (19*.*35%)*			*N = 87 (17*.*72%)*			*N = 97 (19*.*76%)*			*Total Number = 491*			
U18 Male**	*96*	*80*	*65*	*43*	*35*	*46*	*52*	*42*	*39*	*41*	*48*	*36*	*62*.*53*	*<0*.*01*	1.93	-0.74
	*N = 241 (38*.*68%)*			*N = 124 (19*.*90%)*			*N = 133 (21*.*35%)*			*N = 125 (20*.*06%)*			*Total Number = 623*			
Total Male**	***202***	***139***	***112***	***74***	*60*	***85***	***79***	***75***	***66***	***76***	***82***	***64***	***145*.*79***	***<0*.*01***	***2*.*04***	***-0*.*70***
	***N = 453(40*.*66%)***			***N = 219 (19*.*66%)***			***N = 220 (19*.*75%)***			***N = 222(19*.*93%)***			***Total Number = 1114***			
Adult Female*	*49*	*36*	*43*	*36*	*28*	*23*	*38*	*25*	*34*	*30*	*32*	*26*	*11*.*06*	*<0*.*05*	*1*.*46*	*-0*.*63*
	*N = 128 (32*.*00%)*			*N = 87 (21*.*75%)*			*N = 97 (24*.*25%)*			*N = 88 (22*.*00%)*			*Total Number = 400*			
U18 Female**	*71*	*56*	*61*	*40*	*39*	*38*	*32*	*42*	*36*	*52*	*41*	*29*	*29*.*66*	*<0*.*01*	*1*.*52*	*-0*.*67*
	*N = 188 (35*.*07%)*			*N = 117 (21*.*83%)*			*N = 109 (20*.*34%)*			*N = 122 (22*.*76%)*			*Total Number = 537*			
Total Female**	***120***	***92***	***104***	***76***	***67***	***61***	***70***	***67***	***70***	***82***	***73***	***55***	***38*.*11***	***<0*.*01***	***1*.*50***	***-0*.*71***
	***N = 316(33*.*72%)***			***N = 204 (21*.*77%)***			***N = 207(22*.*09%)***			***N = 210 (22*.*41%)***			***Total Number = 937***			
***Total*** (Male+Female)**	*322*	*231*	*216*	*150*	*127*	*146*	*149*	*142*	*136*	*158*	*155*	*119*	*170*.*82*	*<0*.*01*	*1*.*54*	*-0*.*73*
	*N = 769 (37*.*50%)*			*N = 423 (20*.*62%)*			*N = 20*.*62 (20*.*82%)*			*N = 432 (21*.*06%)*			*Total Number = 2051*			

X^2^ = Chi-square test; P = significance

*p<0.05 and

**p<0.01; Cor = Correlation; OR = Odds ratio of Q1 vs. Q4

To gain a better understanding of the impact of the RAE on playing position, we considered the distribution of players’ birthdates across the different positions. From the results presented in Table 2 and Table 3, all four field positions demonstrated RAE (p<0.01) in males. However, only three positions (goalkeeper, defender and midfield) demonstrated RAEs in females (p<0.05, <0.01 and <0.01 respectively).

**Table 2:** The birthdate distribution for player positions in male elite players.

*Position*	*Q1 (%)*	*Q 2 (%)*	*Q3 (%)*	*Q4 (%)*	*Total*	*X*^*2*^	*p*	*V*	*Cor*
GK**	*56 (39*.*7)*	*21 (14*.*9)*	*36 (25*.*5)*	*28 (19*.*9)*	*141*	*19*.*49*	*<0*.*01*	*0*.*22*	*-0*.*59*
DF**	*173 (44*.*8)*	*66 (17*.*1)*	*69 (17*.*9)*	*78 (20*.*2)*	*386*	*81*.*67*	*<0*.*01*	*0*.*27*	*-0*.*86*
MF**	*167 (38*.*1)*	*100 (22*.*8)*	*89 (20*.*3)*	*82 (18*.*7)*	*438*	*41*.*76*	*<0*.*01*	*0*.*18*	*-0*.*67*
FW**	*56 (37*.*6)*	*32 (21*.*5)*	*26 (17*.*4)*	*35 (23*.*5)*	*149*	*13*.*71*	*<0*.*01*	*0*.*18*	*-0*.*68*

GK = goalkeeper; DF = defender; MF = midfielder; FW = forward; X^2^ = Chi-square test; p = significance; Cor = Correlation; V = Cramer’s V(*/**/***:small/medium/large effect size)

**Table 3:** The birthdate distribution for player positions in female elite players.

*Position*	*Q1 (%)*	*Q 2 (%)*	*Q3 (%)*	*Q4 (%)*	*Total*	*X*^*2*^	*p*	*V*	*Cor*
GK**	*43 (38*.*7)*	*22 (19*.*8)*	*22 (19*.*8)*	*24 (21*.*6)*	*111*	*11*.*27*	*<0*.*05*	*0*.*18*	*-0*.*72*
DF*	*108 (33*.*1)*	*94 (28*.*8)*	*59 (18*.*1)*	*65 (19*.*9)*	*326*	*20*.*09*	*<0*.*01*	*0*.*14*	*-0*.*91*
MF*	*124 (31*.*2)*	*91 (24*.*3)*	*66 (17*.*6)*	*93 (24*.*9)*	*374*	*18*.*11*	*<0*.*01*	*0*.*13*	*-0*.*64*
FW*	*41 (32*.*5)*	*33 (26*.*2)*	*24 (19*.*0)*	*28 (22*.*2)*	*126*	*5*.*11*	*>0*.*05*	*0*.*12*	*-0*.*86*

GK = goalkeeper; DF = defender; MF = midfielder; FW = forward; X^2^ = Chi-square test; p = significance; Cor = Correlation; V = Cramer’s V(*/**/***:small/medium/large effect size)

The data in Tables 2 and 3 indicate associations of varying effect size between playing position and relative age in this sample. According to the effect sizes, the RAE was strong for all positions in male players (V>0.17). In contrast, in the female players, there were strong effects in goalkeepers (V = 0.18) along with relatively small effects in defenders and midfielders (V = 0.14 and 0.13 respectively). The effect in forwards was not statistically significant but was generally the same size as in defenders and midfielders (i.e., V = 0.12).

## Discussion

In order to increase our understanding of the impact of social and cultural factors in promoting the RAE, this study examined RAEs in elite male and female Chinese soccer players who participated in the 13^th^ China National Games in 2017. There was a clear RAE reflecting an overrepresentation of players born in the first quartile of the year. More specifically, the percentage of male players born in the first half of the selection year (Q1 and Q2) was 62.53% and significantly greater than the 37.48% observed in the second half (Q3 and Q4). These findings are in line with previous examinations of the RAE in soccer [12, 21, 25, 28], providing consistent evidence for the existence of RAEs in soccer across the world. Soccer is the most popular team sport in the world, and one that emphasizes body size and physical strength as determinants of performance [18]. As a result, players born early in a cohort are more likely to possess better scores on measures of skill and physical fitness, which advantage them over their peers in the same age group [3, 32]. Consequently, younger players who may possess high potential are less likely to be selected for elite teams [43] and more likely to drop out [44].

While there has been considerable research on the RAE in male sports, particularly soccer, there has been much less conducted on female sports. In the current study, we also found significant RAEs in elite Chinese female soccer players, which were in line with previous results from France and Spain [45, 46] and consistent with prior work demonstrating RAEs in female sports are generally not as large as in male sports [47]. While the exact cause of these differences is not known, the exercise intensity in female sports is lower compared to male sports [48]. Several authors have noted that RAEs in female sports reduce as competitive level increases, especially in the German handball, US and French soccer [9, 31, 45]. As for the slight decline of significance of the RAE in adult females, it could be explained by the interaction between biological and maturational differences on the one hand and societal influences on the other [48]. For example, along with increasing of age, athletic performance of female players shortly after menarche may reduce some of the physiological benefits of being born early in the selection year [48]. As well, the pressure to conform to a socially constructed gender role could make early maturing females less motivated to achieve a high level of performance because Chinese society does not value female athletes’ accomplishments in the same way it does for males [49].

To better to understand the RAE in this sample, we also examined how the size of the effect varied across different playing positions. RAEs appeared to be larger for positions that are more physically intensive (i.e., in goalkeepers and central defenders) [50]. This corroborates previous research suggesting the magnitude of RAEs was related to physical demands and players’ positions in ice-hockey [51] and handball [9]. If we focus on the statistically significant results, there were RAEs in every position, with the exception of female forwards. On the one hand, the effect in female forwards was roughly the same size as for defenders and midfielders so we are cautious not too make too much out of this finding. On the other hand, this observation is in line with previous studies showing defenders and goalkeepers are overrepresented by players who were born in the first half of the year [26]. In particular, the position of defender represents the only position in which the RAE has been observed consistently, likely because of the higher physical demands and more advantaged morphological size for this position on the field [52]. Similarly, some have claimed that the RAE for the position of the goalkeeper would be similar to the position of the defender because of the same morphological demands [52]. In our results, there was a relatively large effect size for goalkeepers in both the males and females compared to the other positions. Although the RAE was still statistically significant for the other two positions (defenders and midfielders) in females, the effective size was relatively small. This may be due to the decrements in physical fitness after puberty, which is in line with the assumption that post-pubescent females generally have shorter lower limbs in overall proportion and develop wide hips compared to their previous physique leading to the disadvantages in performing motor skills, such as jumping and running [48]. Our results also support previous work indicating the size of the RAE for forwards in youth soccer was smaller than for midfielders, defenders and goalkeepers [26].

Our results highlight that even in the unique cultural and social system of Chinese sport, RAEs persist and thrive. Given the pervasiveness of RAEs throughout competitive sport, several researchers have forwarded recommendations to reduce or eliminate the effect, such as replacing chronological age with biological age [53,54], changing or rotating the selection date [55], improving the coaches’ opinions of the talented players [55–57] and using the age-ordered shirts during selection [58]. However, in the current study we only explored the existence of RAEs in this population and were unable to identify the causes of these effects due to limitations (e.g., the large population and the inaccuracy of the birthdates in rural areas) in the data collection for elite players in China.

## Limitations

Despite the strengths of this study (e.g., unique sample and context, large sample), there were several limitations. First, this study examined RAEs only in participants at the China National Games in 2017, which may not accurately represent the overall population of elite Chinese youth soccer players. Second, collapsing the two-year age bands is certainly a limitation of our study. This issue merits a debate among RAE researchers that has already been initiated by Steingrover et al. (2017).

## Conclusions

Previous research has indicated the Chinese ‘one-child policy’ resulted in a decrease of both the national birth rate of new born babies [39] and number of sport participants [40]. Interestingly, a clear RAE remains in elite Chinese soccer across gender and age groups (U18, U20 male and U18 female). RAEs were also observed in certain playing positions of the male players and female players. Goalkeepers and defenders demonstrated the strongest RAEs in male players while goalkeepers had the largest effects in females. Greater attention to RAEs throughout player development systems is important to reduce the negative consequences associated with these effects. Greater attention to RAEs throughout player development systems is important to reduce the negative consequences associated with these effects. And future researchers should also consider the correlation between the RAE and the individual’s maturity status, because the maturation is one of the key factors to the talent selection both in male and female players [30].
